# Rats, Neuregulins and Radical Prostatectomy: A Conceptual Overview

**DOI:** 10.3390/jcm12062208

**Published:** 2023-03-13

**Authors:** Dorin Novacescu, Alexandru Nesiu, Razvan Bardan, Silviu Constantin Latcu, Vlad Filodel Dema, Alexei Croitor, Marius Raica, Talida Georgiana Cut, James Walter, Alin Adrian Cumpanas

**Affiliations:** 1 Doctoral School, Victor Babes University of Medicine and Pharmacy Timisoara, E. Murgu Square, Nr. 2, 300041 Timisoara, Romania; 2 Department Medicine, Discipline of Urology, Vasile Goldiş Western University, Liviu Rebreanu Boulevard, Nr. 86, 310414 Arad, Romania; 3 Department XV, Discipline of Urology, Victor Babes University of Medicine and Pharmacy Timisoara, E. Murgu Square, Nr. 2, 300041 Timisoara, Romania; 4 Department II, Discipline of Histology, Victor Babes University of Medicine and Pharmacy Timisoara, E. Murgu Square, Nr. 2, 300041 Timisoara, Romania; 5 Angiogenesis Research Center, Victor Babes University of Medicine and Pharmacy Timisoara, E. Murgu Square, Nr. 2, 300041 Timisoara, Romania; 6 Department XIII, Discipline of Infectious Diseases, Victor Babes University of Medicine and Pharmacy Timisoara, E. Murgu Square, Nr. 2, 300041 Timisoara, Romania; 7 Center for Ethics in Human Genetic Identifications, Victor Babes University of Medicine and Pharmacy Timisoara, E. Murgu Square, Nr. 2, 300041 Timisoara, Romania; 8 Emeritus, Department of Urology, Loyola Medical Center, Maywood, IL 60153, USA

**Keywords:** men’s sexual health, erectile dysfunction, radical prostatectomy, cavernous nerve injury, rat model, neuregulins (NRGs), neuregulin-1β3 type II, glial growth factor 2 (GGF2), erectile function recovery, male reproductive dysfunction

## Abstract

In the contemporary era of early detection, with mostly curative initial treatment for prostate cancer (PC), mortality rates have significantly diminished. In addition, mean age at initial PC diagnosis has decreased. Despite technical advancements, the probability of erectile function (EF) recovery post radical prostatectomy (RP) has not significantly changed throughout the last decade. Due to virtually unavoidable intraoperative cavernous nerve (CN) lesions and operations with younger patients, post-RP erectile dysfunction (ED) has now begun affecting these younger patients. To address this pervasive limitation, a plethora of CN lesion animal model investigations have analyzed the use of systemic/local treatments for EF recovery post-RP. Most promisingly, neuregulins (NRGs) have demonstrated neurotrophic effects in both neurodegenerative disease and peripheral nerve injury models. Recently, glial growth factor 2 (GGF2) has demonstrated far superior, dose-dependent, neuroprotective/restorative effects in the CN injury rat model, as compared to previous therapeutic counterparts. Although potentially impactful, these initial findings remain limited and under-investigated. In an effort to aid clinicians, our paper reviews post-RP ED pathogenesis and currently available therapeutic tools. To stimulate further experimentation, a standardized preparation protocol and in-depth analysis of applications for the CN injury rat model is provided. Lastly, we report on NRGs, such as GGF2, and their potentially revolutionary clinical applications, in hopes of identifying relevant future research directions.

## 1. Introduction

Prostate cancer (PC) is the second most frequent malignancy (after lung cancer) in men worldwide, with continuously increasing incidence rates. Currently accounting for ~3.8% of all deaths caused by cancer in men, prostate cancer represents the fifth leading cause of death worldwide [1]. Important developments for urological malignancies in general [2,3,4], and PC in particular, include improved diagnostic technologies and fundamental scientific understanding of pathogenesis, as well as steadily evolving clinical tools for screening/early detection and risk stratification/therapeutic decision-making. In addition, superior and highly specific clinical methods are available for PC diagnosis, which include Prostate Specific Antigen (PSA) screening, multi-parametric magnetic resonance imaging (mpMRI), PSA isoforms, and micro ribonucleic acid (microRNA). These methods have greatly facilitated early detection [5]. Subsequently, mortality rates were significantly diminished, especially in developed countries (~10.1/100,000 people in Western Europe in 2018 [1]), where these emerging clinical tools were more swiftly integrated. This improvement in oncological outcomes has resulted from more organ-confined disease at initial diagnosis and allowing for immediate curative treatment, i.e., radical surgery or radiotherapy [6]. Unfortunately, only modest improvements have been achieved regarding surgical-treatment-associated morbidity. Postoperative functional complications remain quite frequent and still severely impact quality of life for PC survivors [7].

Radical prostatectomy (RP) is the most widely recommended curative therapeutic procedure for patients with intermediate-to-high-risk prostate cancer and a life expectancy of at least 10 years [8]. Despite the advancements in surgical techniques and postoperative management, no significant change has been observed regarding the probability of erectile function (EF) recovery after surgery over the last decade [9]. Simultaneously, as we entered the current era of mostly curative therapeutic management of PC, mean age at initial diagnosis of PC has decreased, while overall life expectancy has steadily increased. Therefore, RP-associated morbidity, due to intraoperative lesions of the cavernous nerve (CN) plexus, has now begun affecting much younger patients. Thus, currently, especially when tumor loco-regional extension allows for the use of a nerve-sparing RP technique, postoperative functional recovery has become the most important surgical goal. Due to the predisposing inherent regional pelvic anatomy, even in the hands of the most experienced surgeon, regardless of surgical technique or approach used, a certain degree of CN damage usually occurs during RP [10]. Additional strategies are required to improve EF outcomes after RP, seeing as contemporary treatment modalities, i.e., penile rehabilitation protocols, only provide a quicker recovery of EF, not a better rate of recovery overall [11]. Active research, aiming to better comprehend and hopefully identify a curative treatment for CN-lesion-related pathology, is currently ongoing. Even so, for the moment, the pervasive need for a significant improvement in the clinical management of post-RP ED remains unaddressed and requires further analysis.

To further pursue the overarching scientific goal of developing novel therapeutic and/or prophylactic clinical tools, aiming to reshape the persistently inefficient landscape of neuronal-injury-related urinary pathologies in general [12], and RP-associated morbidity in particular, multiple animal models have been developed and utilized in various experimental settings. Although imperfect, the physiological similarities between many animal species and humans allow for the replication and analysis of certain key biological processes involved in the pathogenesis of diseases that affect humans. Furthermore, these models can be much more easily and invasively manipulated, i.e., genetically modified, exposed to novel experimental molecules, and/or subjected to extensive surgical procedures, facilitating a timelier and more ethical and cost-effective exploration of specific interventions and their subsequent effects within a precisely controlled environment. Moreover, experimental animal model study designs usually provide well-documented standardized methodological protocols, meaning that these investigations offer a high level of repeatability, increasing the reliability and reproducibility of the results [13].

A plethora of studies utilizing animal models of CN lesions have been published, analyzing the use of systemic and local treatment modalities to aid in EF recovery. Most promisingly, neuregulins (NRGs) have demonstrated their neurotrophic effects, both regarding central nervous system degenerative disease, but also in the context of peripheral nerve injury. Recently, a seminal investigation, utilizing the well-established and generally preferred rat model of CN lesions, has provided consistent experimental data, supporting the use of neuregulin-1β3 type II, i.e., glial growth factor 2 (GGF2), as a novel systemic treatment modality, with far superior dose-dependent neuroprotective and neurorestorative effects as compared to previous therapeutic counterparts analyzed [14]. However, these initial findings, although potentially impactful, remain limited and inconsistent, as protocols are still unstandardized regarding GGF2 dosing and posology, i.e., combined intravenous administration before injury and local administration at the time of surgery.

The overarching aim of the current paper is to provide further aid within the cumbersome process of developing an integrative and clinically impactful prophylactic/restorative strategy for post-RP ED management. Initially, authors establish the contemporary scientific background regarding post-RP ED pathogenesis, while also reviewing the disease-specific therapeutic strategies currently available to clinicians. Subsequently, the currently available experimental data, regarding emerging therapeutic agents and their reported response rates, within CN lesion animal models, were analyzed. Furthermore, in order to clarify lingering methodological inconsistencies among currently available CN lesion animal model preparation protocols, an in-depth analysis of the most commonly used, and generally preferred, rat model of CN lesions is provided. In the interest of methodological homogeneity, an integrative standardized surgical preparation protocol was elaborated to serve as a reference for further study designs. Lastly, the recent and extremely promising neuroprotective and/or neuroregenerative effects of NRGs are reviewed, in an attempt to further explore novel relevant future research directions and their potentially impactful clinical applications.

## 2. Pathogenesis of Radical-Prostatectomy-Associated Morbidity

The oncological goal of RP is to achieve a complete removal of the cancerous prostate tissue in order to cure the patient of PC. The surgical procedure involves removing the entire prostate, with its capsule intact, alongside the seminal vesicles and distal vas deferens, followed by vesico-urethral anastomosis. Additionally, bilateral ilio-obturatory pelvic lymphadenectomy may also be required in high-risk cases. Surgical approaches have greatly evolved, from open perineal/retropubic approaches to the newer laparoscopic and robot-assisted techniques. Importantly, the increased complexity of the procedure, the associated reconstructive surgical challenges, and the inherent anatomical particularities of the pelvic region greatly facilitate the occurrence of significant postoperative complications. Thus, the overall goal of this surgery is not only radical oncological excision, but also the preservation of the patient’s quality of life, to the greatest extent possible [15].

Essentially, there are two major types of complications, which occur after RP, in the late postoperative setting, namely, obstructive, i.e., anastomosis stenosis, urethral strictures, meatal stenosis; and functional, i.e., impotence and incontinence. Regarding obstructive complications, multiple endoscopic treatment modalities are available and offer a reasonable rate of success. Confoundingly, regarding post-RP functional complications, even though in recent years, mainly due to technical and conceptual advancements in RP surgical strategy, the rate of urinary incontinence has steadily decreased, post-RP ED remains disproportionally prevalent [16]. Post-RP ED usually occurs due to the almost unavoidable intraoperative damage of the regional autonomic innervation, especially during dorsolateral prostatic dissection. This damage may be mechanically induced (nerve division, crush, or stretching), or secondary to thermal damage, ischemia, and/or local inflammatory responses [14], with subsequent Wallerian degeneration of injured nerve fibers and dysregulation of neuromodulated tissue oxygenation. These complications are more difficult to treat and, until now, no specific curative treatment protocol has been developed.

Conversely, the ever-growing arsenal of both biomarkers and modern multimodal imaging [17] has indeed greatly facilitated PC diagnosis, clinical staging, and risk stratification, as well as RP surgical planning. Specifically, mpMRI and targeted biopsies have revolutionized the diagnosis and management of PC. MpMRI, a combination of anatomical imaging techniques (T2-weighted imaging) and functional imaging techniques (dynamic contrast-enhanced MRI, diffusion-weighted MRI, and magnetic resonance spectroscopy), offer superior imaging capabilities that enable the accurate identification of suspicious areas in the prostate gland, thus reducing unnecessary biopsies and increasing the detection of clinically significant PC [18]. Targeted biopsies, guided by mpMRI, are more accurate in detecting PC lesions and have a higher yield of clinically significant disease than traditional systematic biopsies [19,20,21]. Furthermore, mpMRI can aid surgeons during robotic RP by providing detailed anatomical information on the location and extent of the cancer within the prostate gland and accurately predicting the location and extent of extraprostatic extension, seminal vesicle invasion, and lymph node metastasis, which are all important factors to consider when planning the nerve-sparing technique (i.e., intra, inter, or extrafascial nerve sparing) [22,23,24].

### 2.1. Predisposing Anatomic Considerations

In human males, the inferior hypogastric plexus, also known as also known as the pelvic plexus, represents the central innervation hub of the pelvic cavity. Constituting a complex network of nerves, located near the base of the bladder, anterior to the rectum, the inferior hypogastric plexus emerges as a result of the convergence and intermingling of sympathetic fibers from the lumbar sympathetic ganglia and parasympathetic fibers from the sacral spinal cord, which provide autonomic innervation to the main pelvic viscera, i.e., the bladder, rectum, and male reproductive organs—seminal vesicles, vas deferens, prostate, and penis. Additionally, and distinctly, this plexus also contributes to the sensory innervation of the perineum and anus [25].

**Figure 1:**
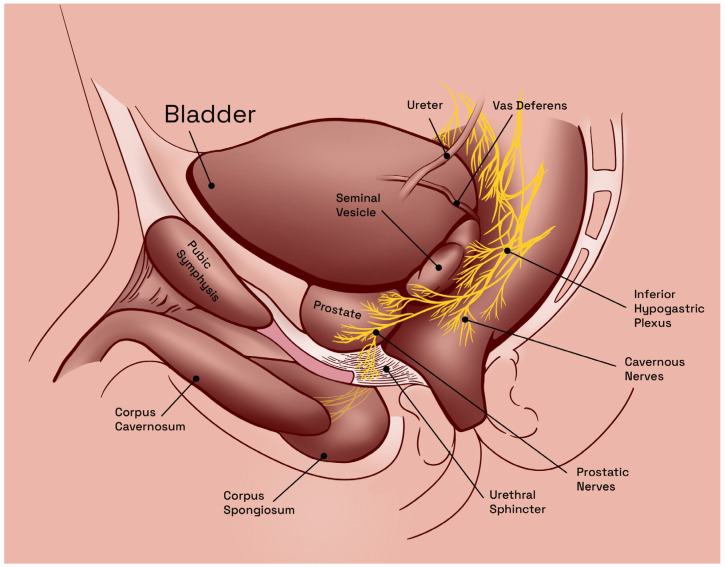
Anatomy of the male pelvic cavity in humans, sagittal view. Illustration adapted from Arthur L. Burnett [26].

The anatomic relationships of the inferior hypogastric plexus are complex and involve multiple structures within the pelvic region (Figure 1). Some of the key anatomic relationships of the inferior hypogastric plexus include proximity to the bladder base and postero-inferior bladder wall, anterior aspect of the rectum, and proximity to major blood vessels (aorta and the internal iliac arteries) [25]. Overall, the anatomic relationships of the inferior hypogastric plexus are complex and involve multiple structures within the pelvic region. Understanding these relationships is important for the diagnosis and treatment of a variety of pelvic conditions.

**Figure 2:**
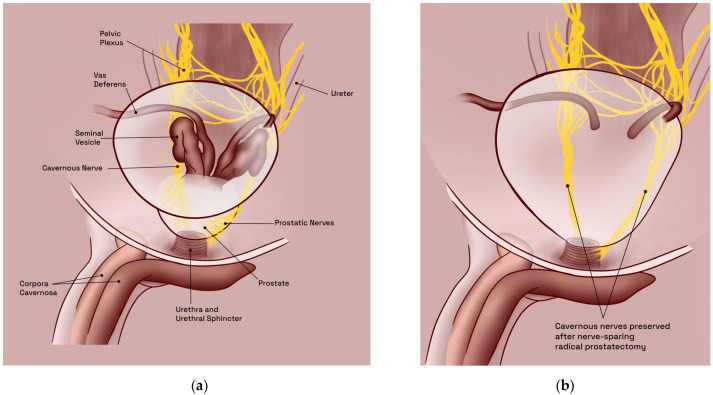
Comparative illustrations of male pelvic anatomy in humans, oblique view: (**a**) basic physiological layout; (**b**) post bilateral nerve-sparing radical prostatectomy. Illustrations adapted from Arthur L. Burnett [26].

The CNs are a pair of parasympathetic nerves, which arise from the inferior hypogastric (or pelvic) plexus and run along the lateral aspect of the prostate, entering the corpus cavernosum at the base of the penis (Figure 2a). They are responsible for the regulation of blood flow into the erectile tissue and play a crucial role in the physiological mechanism of sexual arousal and erection. More specifically, the CNs comprise small myelinated and unmyelinated nerve fibers, responsible for the regulation of parietal smooth muscle tonus within the penile blood vessels, and are thus able to increase the blood flow to the corpus cavernosum, resulting in penile erection [27]. Thus, extensive damage to the CNs will result in ED. Therefore, the anatomy of the CNs is crucial for normal sexual function and their proper functioning is essential for maintaining sexual health.

Pelvic surgery in general, be it for prostate, bladder, or colorectal malignancies, commonly results in a high incidence of ED due to trauma of the CNs, the principal autonomic innervation of the penis [14]. Notwithstanding recent advancements in both surgical techniques (nerve-sparing procedures) and equipment (robot-assisted approach), these types of neurological lesions remain prevalent and virtually unavoidable, with less than 40% of patients regaining EF, sufficient for sexual intercourse, following bilateral CN-sparing surgery [14,28,29] (Figure 2b). However, regardless of surgical technique and/or approach used, the incidence of post-RP ED remains high and represents a serious problem, especially in young patients [30]. Thus, in the era of early detection, with mostly curative initial treatment for prostate cancer, age at diagnosis has decreased, while life expectancy has steadily increased. Postoperative functional recovery has become the most important surgical goal when performing RP.

### 2.2. Pathophysiology of Erectile Dysfunction following Nerve-Sparing Radical Prostatectomy

In the physiological setting, the pelvic plexus is the origin of innervation for erection. The CNs contain parasympathetic and sympathetic fibers, with the proximal ganglionic area containing both myelinated and unmyelinated fibers. This conformation gradually changes distally, with fewer myelinated fibers being represented, until the point of crural entry, where the CNs are almost exclusively composed of unmyelinated axons. Therefore, unmyelinated axons represent the part of CN fibers which provide the neurotransmitters for penile innervation, the most important one being nitric oxide (NO) [14,31,32]. Sexual stimulation produces NO release at the level of these fibers, which will then induce an increase in oxygenated blood flow to the erectile tissue, by relaxing parietal arterial and arteriolar smooth muscle fibers [33]. The increased blood flow produces distension forces acting upon the endothelium, leading to a sustained nitric oxide synthase (eNOS) release from endothelial cells. This mechanism is crucial for erection prior to intercourse as well as the long-term maintenance of corporal health [34]. Even in the hands of the most experienced surgeon, regardless of the surgical technique or approach used, a certain degree of CN damage occurs during RP [35]. This nerve injury can result from retraction injury during surgery, electrocautery damage, neural vasculature disruption, or rampant local inflammation post compression trauma [36]. This surgical trauma is the causal agent of impaired parasympathetic penile function, manifested as ED [37].

**Figure 3:**
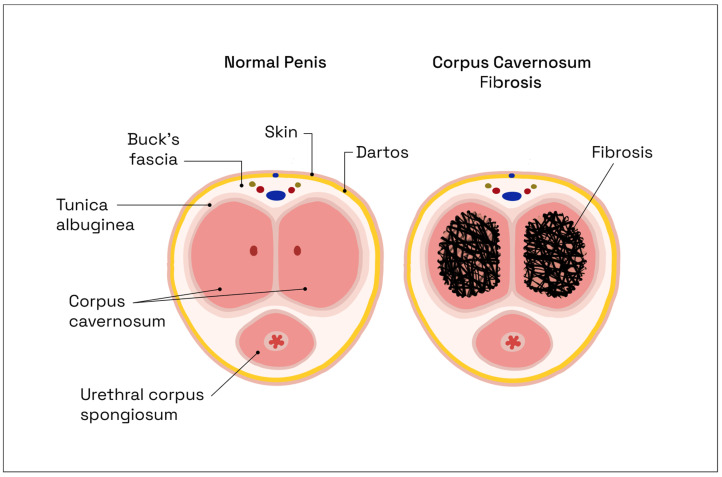
Comparative axial anatomic sections of the penis, illustrating the progressive process of fibrotic connective tissue buildup within the corpus cavernosum. Illustrations adapted from Milenkovic et al. [38].

Postoperative nerve damage will induce tissue hypoxia, which in turn leads to a decrease in NO production, thus creating a vicious circle, while also diminishing, simultaneously, the production of anti-fibrotic protective mediators [30], resulting in fibrous connective tissue buildup with smooth muscle apoptosis (Figure 3). These fibrotic changes, which are irreversible, diminish tissue elasticity and make penile expansion difficult [35]. Thus, acute intraoperative lesions of the CNs initiate a chronic, irreversible, vicious circle of structural and metabolic modifications within the cavernous tissue.

**Figure 4:**
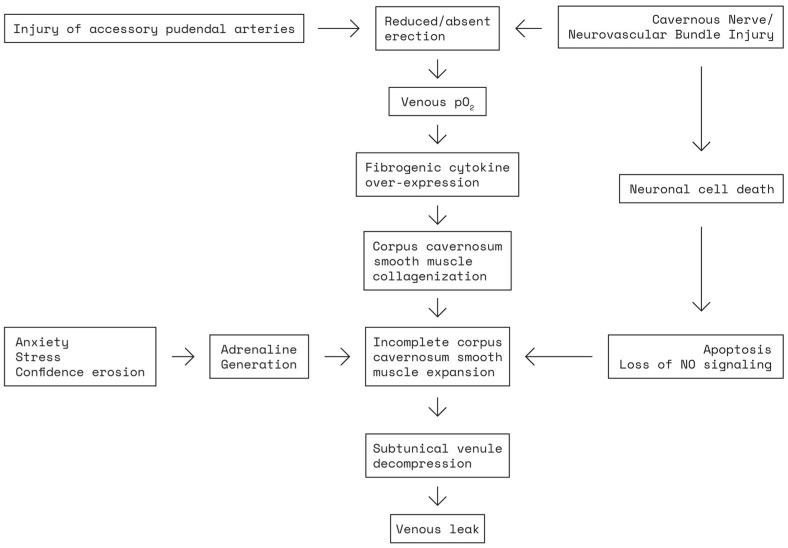
Pathophysiology of post radical prostatectomy erectile dysfunction. pO_2_ = partial pressure of oxygen; adapted from Mulhall et al. [40].

In addition to vaso-occlusive disease, it is possible that the deposition of collagen is due to cellular apoptosis of smooth muscle (not of the endothelium), particularly in the subtunical area, causing dysfunction of the veno-occlusive mechanism of the corpus cavernosum. These mechanisms underlie the etiology of the massive corporeal venous leaks that follow [39], thus adding a secondary venogenic component of ED (Figure 4). Therefore, while the occasional use of erectogenic pharmacotherapy will likely produce a transient erection, especially early after surgery, an underlying long-term deterioration of the normal physiologic processes involved in penile erection is already underway [39].

In summary, post-RP ED is caused by interference with the neurological mechanisms that facilitate cavernosal oxygenation, leading to fibrosis. A timely re-establishment of tissue oxygenation via neurologically modulated mechanisms is paramount. Currently available management options for post-RP ED all share the same rationale of re-establishing tissue oxygenation, but do not address the causal issue, i.e., CN damage, only the consequences in a later, postoperative setting, making clinical applications, such as standardized perioperative systemic prophylaxis of CN lesions, a very desirable outcome.

## 3. Current Treatment Options for Post-Radical-Prostatectomy Erectile Dysfunction

Despite the existence of multiple therapeutic modalities for ED (phosphodiesterase 5 inhibitors, vacuum erection devices, intraurethral therapy with prostaglandin E1 analog suppositories, and intracavernosal injections with TriMix—papaverine, phentolamine, and PGE1 (penile prosthesis implantation)), a proper, irrefutably validated, clinical guideline for sequential post-RP ED therapeutic management has not yet been standardized [30]. Moskovic et al. have previously elaborated a penile rehabilitation program [39], in an attempt to systematize the therapeutic approach and limit cavernosal fibrosis, with PDE-5 inhibitors as the first-line treatment for post-RP ED, and vacuum erection devices, local delivery systems for PGE1 analog, and intracavernosal injections as alternatives for non-responders to PDE-5 inhibitors. In this model, penile prosthesis implantation represents the final solution, reserved solely for those medical treatment refractory patients exhibiting a persistent failure to respond to maximal conservatory therapy after a two-year evaluation period [39]. Still, functional results are modest and postoperative ED remains a major cause of morbidity after RP.

The complexity of the mechanisms predisposing post-RP patients to ED suggests the need for multiple points of intervention to prevent the development of ED in these patients, in the absence of a causal prophylaxis strategy for intraoperative CN injury. Most importantly, it has been stated that tissue oxygenation may reduce the prevalence of chronic inflammation and cavernosal fibrosis [41], albeit, interestingly, hyperbaric oxygen therapy in an animal model of CN injury has not been shown to significantly reverse or minimize this process, suggesting that there are multiple mechanisms involved [41]. Secondarily, cytokine mediators of tissue fibrosis and inflammation represent targets for pharmacotherapy, aimed at preserving cavernosal tissue integrity. The concept of erectile preservation is premised on minimizing the factors that impair long-term erectile function, although this strategy does not represent a curative etiological treatment, serving solely the purpose of limiting the damage, not of preventing it from happening altogether. Therefore, at the moment, therapeutic strategies targeting the aforementioned mechanisms, consecutive to CN damage, are still failing to provide patients with optimal functional outcomes [39].

A 2019 literature review, conducted by Capogrosso et al., concluded that despite the advancements in surgical techniques and postoperative management of patients treated with RP, no significant change was observed regarding the probability of EF recovery after surgery over the last decade, in a single high-volume center [9]. Additional strategies are required to improve EF outcomes after RP, seeing as penile rehabilitation only provides a quicker recovery of EF, not a better rate of recovery overall. Active research is ongoing regarding CN-lesion-related pathology, as the burning need for an improved treatment strategy for ED remains unaddressed.

Recently, utilizing the CN lesion rat model, a novel therapeutic approach was investigated in the experimental setting, addressing these intraoperative CN lesions in a prophylactic, neuroprotective manner, by establishing a standardized protocol for perioperative neuregulin-based systemic treatment with GGF2 [14]. Hopefully, the preoperative administration of GGF2 will further demonstrate its neurotrophic effect, making it harder for clinically significant CN lesions to occur, while also reconfirming the neurorestorative properties of GGF2 in the postoperative setting, with a clearer quantification of the molecular mechanisms involved. Thus, the prophylactic intervention would specifically address the cause (nerve damage with subsequent Wallerian degeneration) and not the consecutive metabolic effects, curing the disease before it even occurs.

## 4. The Cavernous Nerve Lesion Rat Model

The rat model of CN lesions was firstly described by Quinlan et al. in 1989 [42] and, throughout the following decades, a plethora of studies have been released [43,44,45], utilizing this animal model as a means of analyzing the effects of various therapeutic principles, aiming to alleviate ED, post autonomic nervous lesions, i.e., usually referring to the clinical context of RP, which is replicated through this animal model in a controlled experimental setting.

### 4.1. Advantages and Limitations of the Cavernous Nerve Lesion Rat Model

The aforementioned CN lesion rat model represent the most prevalently used and generally preferred animal model for replicating the clinical context of post-RP ED in humans, as it offers several advantages over other existing models. Firstly, and most importantly, the anatomy of this particular model is inherently favorable for CN preparation, controlled injury, and interventional therapeutic experimentation. More specifically, in male rats, the CN can be isolated bilaterally within the pelvic cavity, as a morphologically distinct, individual nerve, rather than a plexus of autonomic nerves as seen in humans. This single nerve anatomy facilitates identification, dissection, and manipulation [42]. Moreover, nerve conduction can be easily quantified in an objective manner by corporal body cannulation and subsequent intra-corporeal erectile pressure recordings. Thus, CN-injury-related conduction deficits, as well as the functional effects of restorative interventions, can also be objectively assessed by comparative analysis of the modifications in intra-corporeal pressure (ICP) values in relation to mean arterial pressure (MAP), achieved through standardized electrostimulation of the CNs, before and after injury induction and therapeutic intervention, respectively. Finally, the cost-effectiveness of rat models constitutes an equally important practical advantage [46].

Conversely, in contrast to the previously discussed advantages over other existing animal models, the CN lesion rat model also incurs a number of experimental limitations. First of all, an overwhelming majority of previous reports, focusing on this particular rat model, have generally utilized solely young and healthy animal specimens for CN lesion preparations, whereas, from a conceptual perspective, this design does not accurately reflect the clinical realities of PC incidence [47]. For the most part, PC is still usually diagnosed in older men, with important underlying microangiopathic comorbidities (i.e., diabetes, obesity, alcohol and tobacco addiction, chronic kidney disease, cardiovascular and neurological pathologies), which entail a certain degree of preexisting hypoxic cavernosal modifications [48]. Thus, in this older PC population, the post-RP CN damage will actually have a cumulative effect, by additionally accentuating these pre-existing cavernosal modifications, whereas, in younger, otherwise healthy patients, RP will represent the initial primary inaugural event for cavernosal tissue fibrotic transformation.

**Figure 5:**
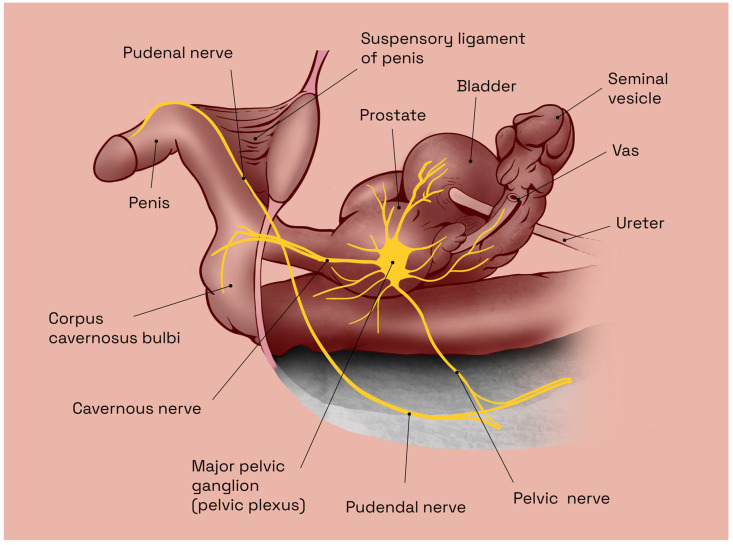
Lateral view of rat model pelvic anatomy. Illustration adapted from Quinlan et al. [34].

Significantly, another inherent limitation of this model was brought to light by Sato et al. (2001) in a groundbreaking paper identifying and characterizing the restorative role of inherent rat ancillary nerve fibers, in the chronic setting, post-CN injury [49]. Central nervous system stimulation, even after bilateral CN transection, showed significant response rates, with sustained increases in ICP values. In contrast, the ICP response was abolished following a complete bilateral pelvic nerve transection, i.e., including ancillary nerve fibers. These nicotinamide-adenine dinucleotide phosphate diaphorase (NADPH-d) fibers, also originating from the major pelvic ganglia (MPG) (Figure 5), have a complementary role to the CNs, pertaining to the autonomic motor innervation of the penis, contributing 40–50% to ICP response after central nervous system stimulation [49]. This may be one of the reasons why, in the absence of significant comorbidities, rats undergoing CN lesions will recover erectile function spontaneously after ~6 months [50]. Another concern, especially in chronic studies, is the insufficient washout period for the treatment administered, which can lead to a misinterpretation of EF recovery due to the continued presence and effects of treatment.

### 4.2. Anatomy and Standard Preparation Protocols for the Cavernous Nerve Rat Model

Anatomical dissections demonstrate a bilateral ganglion lateral to the prostate called the MPG (Figure 5). This ganglion receives input from the pelvic and hypogastric nerves and innervates the pelvic viscera. A large fiber from the major pelvic ganglion courses along the urethra and innervates the corpus cavernosum, the CN [42].

As practical considerations for rat model preparation, preoperative assessment of both surgical strategy and the availability of necessary equipment are essential for the accuracy of experimental results. The generally preferred rat species is Sprague Dawley rats, usually ~12 weeks old in acute CN injury assessment studies [51]. Inhalation isoflurane anesthesia, for better anesthetic control of the model, in order to maintain adequate blood pressure, is highly recommended. A low-magnification surgical microscope or microsurgery loupes are required for operative field visualization, as well as a microsurgery kit with adequate electrocautery tools. An electric stimulator connected to a two-prawn electrode, 3 mm apart, is seemingly the ideal setup to properly stimulate the CN, proximal to the site of the mechanic lesion. Adequately adjusted pressure transducers and recording equipment, for the accurate documentation of MAP and ICP variations, are essential for the validity of the functional response results [52].

The surgical strategies involved in the practical anatomic preparations of key topographic areas are multi-faceted and must be thoroughly assessed beforehand. Both the bilateral dissection of the CNs, to allow for electrostimulation, as well as the cannulation of the carotid artery and corpus cavernosum, for MAP and ICP measurements, respectively, are usually required for accuracy in experimental design [52]. Regarding the standardization of CN surgical injuries, the crush lesion model is generally preferred in systemic and topical intraoperative treatment experiments, over the more extreme cut lesion models, which require subsequent microneuroraphy.

For rat CN preparations, the animal is put under general anesthesia and fastened to a test tube rack, in the supine position. A 3–4 cm incision lower midline incision is made, taking care to avoid the deep dorsal vein of the penis. The bladder is located underneath the divided muscles of the abdominal wall, grasped with Allis forceps and mobilized through dissection. Any adhesions to the abdominal wall are divided. The dissection advances caudally and the bladder and prostate are mobilized laterally, to expose the seminal vesicle and vas deferens. The vas deferens is then dissected, and mobilized superiorly, in order to allow visualization of the dorsal aspect of the prostate. The area between the vas deferens and the dorsal lobe of the prostate is then cleared of any overlying fatty tissue in order to expose the CN (Figure 5). Blunt dissection is used to clear adventitial layers of the dorsal aspect of the prostate. Close to the prostate lie pelvic veins, which need to be pushed off the gland with care in order to avoid bleeding. The MPG is located cephalad, on the lateral aspect of the prostate (Figure 5). The CN is shown arising from the MPG and running along the prostate, towards the urethra. An overlying blood vessel is present and can usually be avoided. After initial stimulation for baseline ICP, the nerve lesion is performed [52,53,54].

For obtaining ICP measurements, the penis is circumcised, denuded of skin, and separated from surrounding tissue. A space is developed around the bulbospongiosus muscle. A self-retaining retractor is useful at this stage. The diameter of the penis is small, therefore accurate placement of the needle to measure ICP is essential. At its junction with the inferior pubic rami, the ischiocavernous muscle can be clearly identified. This muscle needs to be carefully isolated from the surrounding tissue and underlying tunica albuginea with curved forceps, and then divided to reveal the white tunica. A 21-gauge needle is then passed into the corporal body to allow penile pressure measurements. Due to the small size, it is only necessary to pass the bevel of the needle into the body. To ensure accurate placement, a small amount of heparinized saline can be flushed through the line. Slight penile tumescence should be seen if the needle has been placed correctly [52,53,54].

For obtaining MAP measurements, a transverse incision is made in the neck, midway between the mandible and sternum. The platysma muscle contains submaxillary glands. An incision is made below these glands and a space is developed under the muscle with care, so as not to cause bleeding from superficial veins, which can be controlled with cautery if needed. The strap muscles of the neck can now be visualized. The sternohyoid muscle is incised over the trachea and the space created is developed in order to identify the pulsatile sheath of the carotid artery. The artery is sharply dissected free of the sheath. It is important to separate out the vagus nerve from the artery, as accidentally including it in the following ligatures will have dire hemodynamic consequences. The carotid artery is tied off superiorly and then gently stretched. A second tie is placed, which will be used to secure the tubing. Finally, a bulldog clamp is placed inferiorly and an arteriotomy is performed. Using a 25 g needle with a bent tip, a heparinized polyethylene tube is guided into the artery. The previous ties are used to secure the tube in place and a pressure transducer is connected to obtain MAP [52,53,54].

For attaching the stimulating electrode, curved forceps are used to guide the electrode into place. The electrode is placed at the junction of the cavernous nerve with the major pelvic ganglion, proximal to the lesion site. The first stimulation should show the absence of conduction, i.e., penile pressure increase, confirming the efficacy of the lesion. In the interest of standardization, electrostimulation protocols for CN lesion rat models have been developed. For example, the European Society for Sexual Medicine (ESSM) recommends using the following stimulator settings: pulse duration—0.5–1 ms; frequency—10–20 Hz; duration of stimulation—30–60 s; voltage—2.5–8 V. Rest periods of at least 5–10 min between stimulations are also recommended [47].

After stimulation data are collected, the animal is euthanized. At this stage, if tissue samples need be collected for further morphopathological treatment response assessment, the dorsolateral prostate tissue, along with the MPG and associated CNs, may be removed, as well as the corpus cavernosum. Morphological evaluation may involve using conventional staining techniques, such as Masson’s trichrome stain to evaluate the smooth muscle/collagen ratio in the corpus cavernosum [55], as well as immunohistochemistry, with a variety of possible targets. For example, anti-nNOS antibodies can be used to quantify neuronal nitric oxide synthase content in the corpus cavernosum [56,57]. Additionally, S100 protein immunostaining may be useful for evaluating Schwann cell populations in the MPG [58].

### 4.3. Previous Applications of the Cavernous Nerve Lesion Rat Model in Post Radical Prostatectomy Erectile Dysfunction Experimental Research

For decades now, sustained and still on-going scientific efforts, invested within a plethora of CN lesion rat model experimental studies, have provided significant data regarding the use of various systemic and local treatment modalities attempting to aid with EF recovery post-RP. Among them, sildenafil has been shown to preserve EF when used in the CN crush injury rat model. This therapeutic property appears to be achieved predominantly by enhanced preservation of smooth muscle content and endothelial function, as well as through a reduction of cavernous erectile tissue cellular apoptosis [53].

More recently, Yamashita et al. evaluated the role of IL-6-mediated inflammatory response in the development of post-RP ED by administering Tocilizumab (anti-rat IL-6 antibody) perioperatively in rat model replicas of RP-induced CN lesions. The investigation reports an increased acute phase interleukin(IL)-6 expression in the MPG, post CN injury. Conversely, IL-6 bioactivity inhibition apparently attenuated ED following CN simple dissection (i.e., without overt injury), as reflected by the increased ICP response rates to stimulation. Thus, the suppression of excess inflammatory responses in the acute phase may lead to improvements in ED occurring after nerve-sparing RP [59]. Similarly, Albersen et al. used pentoxifylline—a phosphodiesterase inhibitor, which further down-regulates multiple cytokine pathways involved in nerve degeneration, apoptosis, and fibrosis —to improve EF recovery, enhance nerve regeneration, and preserve the corpus cavernosum microarchitecture after CN crush injury, within the aforementioned rat model [60]. Additionally, a noteworthy investigation by Mulhall et al. used FK506 (Tacrolimus), an immunomodulatory immunophilin-ligand known to prevent axonal degeneration, in a bilateral CN crush lesion rat model and obtained promising results regarding the preservation of EF following CN injury [61]. Unfortunately, in a recent, randomized, double-blind, placebo-controlled follow-up human trial, despite supportive experimental data, tacrolimus failed to demonstrate any superiority over placebo in EF recovery post-RP [62].

Lastly, various local and systemic neuromodulatory agents have been investigated in animal models simulating RP, in an effort to protect or regenerate CN function and facilitate erection recovery, with some findings suggesting potential clinical benefits. These include other immunophilin-ligands, neurotrophins, growth factors, Schwann cell seeded guidance tubes, glial-cell-line-derived neurotrophic factor, sonic hedgehog protein, atypical neurotrophic factors, nerve guides, tissue engineering/stem cell therapy, and gene therapy [63,64,65,66,67]. However, there are no treatments established for either CN neuroprotection or nerve regeneration at the clinical level [66]. Thus, investigative efforts are still ongoing to identify and develop an ideal strategy that effectively preserves CN function in the clinical context of RP [14].

## 5. Neuregulins, GGF2, and Future Therapeutic Perspectives for Post-Radical Prostatectomy Erectile Dysfunction

NRGs are pleiotropic growth factors that influence cell survival, proliferation, differentiation, and organogenesis throughout the body. Their effects are mediated via interactions with the ErbB family of transmembranary receptor protein tyrosine kinases and the subsequent activation of downstream intracellular signaling events [68]. The ErbB family comprises four receptor tyrosine kinases, structurally related to the epidermal growth factor receptor (EGFR), i.e., the first ErbB family member discovered. In humans, the ErbB family includes Her1 (EGFR, ErbB1), Her2 (Neu, ErbB2), Her3 (ErbB3), and Her4 (ErbB4) [69].

In other words, the term NRGs refers to a subgroup of structurally related signaling proteins, able to bind ErbB receptor tyrosine kinases (ErbB2–4), thus mediating a myriad of cellular functions, including survival, proliferation, and differentiation, in both neuronal [70,71,72] and non-neural tissues [73,74,75]. Discovered independently over two decades ago by several different research groups, these peptide growth factors were originally described as neu differentiation factors (NDFs), heregulins, glial growth factors (GGFs), acetylcholine receptor-inducing activity (ARIA), and sensory and motor neuron-derived factor (SMDF), respectively [76,77]. Currently, it has been established that all of these NRG proteins are encoded by the same gene, namely, *NRG-1* [70,78]. In fact, so far, four genes that encode NRGs in vertebrates have been identified, i.e., *NRG-1, NRG-2, NRG-3,* and *NRG-4*. Among them, the *NRG-1* gene is the most well characterized [79].

The entire human NRG-1 gene has been previously sequenced [79]. Due to multiple promotor usage and alternative splicing, *NRG-1* demonstrates transcriptional heterogeneity, generating well over 30 proteic isoforms, which include the above-mentioned NDFs, heregulins, GGFs, ARIA, and SMDF [77,78,79,80]. Multiple splice variants are also produced from each of the *NRG-2*, *NRG-3*, and *NRG-4* genes. The most important portion of the NRG protein, shared by all the isoforms, is the EGF-like domain, which, by itself, is sufficient for receptor binding and the stimulation of intracellular signaling pathways [81]. Hence, this EGF-like domain is both necessary and sufficient for the biological activity of NRG proteins. The alternative splice variants of this domain give rise to α/β transcripts [82].

Furthermore, NRG-1 isoforms have been further classified into six distinct subtypes, based on the variability of their extracellular amino-terminal domains. More specifically, NRG-1 transcripts type I, II, IV, and V have an immunoglobulin-like (IgG-like) domain, which may be followed by a glycosylation-rich region (type I) or a GGF-specific kringle domain (type II). Type III NRG-1 transcripts present the typical extracellular EGF domain, but are unique in containing a cysteine-rich domain that loops back intracellularly, along with its N-terminal sequence. Apparently, all the types of NRG-1 proteins, with the exception of type III, have both transmembranary forms, as well as secreted forms, depending on whether the isoform is initially synthesized as a transmembranary or non-membranary protein [70,77,79]. These conformational differences may prove to be essential in understanding the underlying metabolomics behind the diverse biologic effects of NRGs.

The expression and emerging neurotrophic, neuroprotective, and neuromodulatory roles of NRGs in the central nervous system are well documented [82]. Multiple experimental investigations, focusing especially on central nervous system degenerative diseases, such as multiple sclerosis or Alzheimer’s disease, have already reported relatively promising results [83]. However, the effects of NRGs in the context of peripheral-nerve-damage-associated disease remains currently under-investigated.

Schwann cells are the main glial cells of the peripheral nervous system, essential to the survival and function of neurons. They are involved in the conduction of nervous impulses along axons, nerve development, trophic support for neurons, and nerve regeneration [84]. Prolonged periods of time in which Schwann cells do not contact axons largely account for very poor functional recovery after peripheral nerve injuries, as denervated Schwann cells die by apoptosis or atrophy and do not support axon growth [85]. The *NRG-1* gene plays a crucial role in axon-glial signaling during the development of the peripheral nervous system [70]. Furthermore, this growth factor is increasingly being recognized for its neuroprotective and neurorestorative properties during adulthood, conceivably through mediating signals between axons and Schwann cells, which are required for effective nerve repair [70,73]. Given these neuroprotective effects, NRGs represent an attractive candidate for protecting the CNs and preserving EF after RP.

GGF2, i.e., neuregulin-1β3 type II, a soluble full-length splice variant of the *NRG-1* gene [70], is known to promote axonal integrity. Even so, to the best of our knowledge, there is only one currently available publication investigating the effects of GGF2 on EF following bilateral CN crush injury (BCNCI) in a rat model of RP-associated CN injury. In 2015, Burnett et al. published the aforementioned paper, which proved that GGF2 systemic treatment, starting just before CN injury, promotes the recovery of rat EF, while also preserving unmyelinated nerve fibers in the injured CN. In light of these results, further clinical evaluation of GGF2, as a neuroprotective therapy for pelvic surgeries (including RP), is mandated yet still unaddressed. The ability to deliver GGF2 remotely, outside of the surgical field, and the ability to pre-treat the experimental models before CN injury, are key advantages for potential translations into the clinical arena [14].

As summarized in Table 1, Burnett et al. set up two investigational protocols for GGF2 treatment response assessment following BCNCI. Protocol 1 was designed to replicate a severe CN lesion, with BCNCI being induced with a serrated hemostat, at a constant ‘one-click’ pressure, for 2 min/side [86]. This severe CN lesion rat population was then randomly divided into three groups (8–10 specimens/group), based on GGF2 administration posology, after BCNCI, namely, vehicle without GGF2; 0.5 mgs of GGF2/kg; and 5 mgs of GGF2/kg. A fourth group had sham surgery and was treated with vehicle. In Protocol 2, for moderate CN lesions, BCNCI was induced with a fine-grade hemostat for 3 min [87]. Animals were again randomly divided into three groups (10–12/group), depending on the same GGF2 administration posology, after BCNCI: vehicle without GGF2; 5 mgs of GGF2/kg; and 15 mgs of GGF2/kg. Additionally, the fourth and fifth groups, with 10–12 specimens each, were sham surgery + vehicle and sham surgery + 15 mgs of GGF2/kg, respectively. Sham surgeries were completed by exposing the CNs but not manipulating them [14].

GGF2 and vehicle were administered subcutaneously 24 h prior to BCNCI, 24 h post-BCNCI, and then once weekly until the end of the study period, 5 weeks post-BCNCI. Treatments were performed in a blinded fashion. The timing and doses of GGF2 used in these experiments were within the range used in previous studies [88,89]. Vehicle contained 20 mM sodium acetate, 100 mM sodium sulfate, 1% mannitol, and 100 mM L-arginine (pH 6.5). The effects were quantified after 5 weeks of treatment by in vivo ICP responses to CN electrical stimulation, retrograde fluorogold tracing, and electron microscopy of the CN [14].

Regarding in vivo ICP responses to CN electrical stimulation, the results demonstrated that GGF2 improves EF following both severe and moderate BCNCI, with a return of maximal ICP to ~50% of the uninjured levels for Protocol 1 and almost comparable to levels of sham operated animals in Protocol 2. Intact neurons within the CN were initially assessed by retrograde fluorogold labeling for reference. Following severe BCNCI and vehicle treatment (Protocol 1), the number of fluorogold-labeled cells in the MPG was reduced by almost 80%. Cell counts were significantly increased after treatment with both 0.5 mg/kg and 5 mg/kg of GGF2, by ~50% and 70%, respectively, yet they were still significantly decreased as compared to the sham surgery group. Moreover, representative electron microscopy images of CNs showed more denervated Schwann cells and more degenerating unmyelinated axons in the BCNCI + vehicle group as compared to the sham surgery and GGF2-treated BCNCI groups. These results indicate that GGF2 protects nerve fibers after BCNI, while also preserving unmyelinated nerve fibers in the CNs [14].

Still, there are a few issues that remain unaddressed regarding the effect of GGF2 on the CN lesion rat model. The exact targets for GGF2 and the inherent metabolic mechanisms involved in preserving CN conduction and EF, following CN injury, warrant further investigations. In the context of peripheral nerve injury, in vitro studies suggest the role of NRG-1 type II in the migration of Schwann cells and macrophages, implying a potential role for NRG-1 in the initial stages of Wallerian degeneration [90]. GGF2-induced Schwann cell migration after nerve crush injuries has been attributed to the induction of α5β1 integrin-ErbB2 receptor-FAK complex formation [91]. In addition to nerve cells, multiple other cells, such as endothelial, smooth muscle, epithelial, and cardiac myocytes, express ErbB receptors and respond to NRGs with growth and differentiation. Thus, it is possible that GGF2 may act on different cell types, in the CNs and within the corpus cavernosum, to exert its neuroprotective or neuroregenerative effects. The integrity of the corpus cavernosum following GGF2 treatment is yet to be adequately evaluated, but could be defined by neuronal NOS expression and smooth muscle/collagen ratio within the corpus cavernosum. Furthermore, the immuno-localization of ErbB receptors within the MPG, as well as in the corpus cavernosum, would suggest the possible site of GGF2 action in protecting penile innervation [14].

## 6. Conclusions

Overall, contemporary PC mortality rates have significantly decreased, alongside the mean age at diagnosis. Contrastingly, the probability of EF recovery post-RP has remained constant throughout the past decade. Thus, due to virtually unavoidable intraoperative CN lesions, post-RP ED has now begun affecting much younger patients. Aiming to address RP-associated morbidity, several CN lesion animal models have been developed, yet the rat model, in particular, remains generally preferred, due to its favorable anatomy, low costs, and high degree of standardization and reproducibility. Utilizing this rat model, response rates to various therapeutic principles aiming to facilitate EF recovery post-RP have already been assessed. Recently, NRGs have shown a remarkable potential for the development of ground-breaking clinical applications, demonstrating neurotrophic effects in both neurodegenerative disease and also peripheral nerve injury models. Most importantly, GGF2 has recently demonstrated far superior, dose-dependent, neuroprotective/restorative effects in the BCNCI rat model as compared to previously investigated therapeutic counterparts. Even so, these initial results are still quite limited and lingering issues remain uninvestigated regarding GGF2 tissue targets, molecular pathology, and metabolomics, as well as the ideal administration route and posology. This immense potential urgently mandates further scientific commitment.

## Figures and Tables

**Table 1:** Distribution of CN lesion rat model population, according to CN injury protocol and treatment applied, as reported by Burnett et al. [14].

	Severe BCNCI * (8–10/Group)	Moderate BCNCI (10–12/Group)	Sham Surgery (8–12/Group)
**Treatment** **regiment**	0.5 mg/kg GGF2 **	0.5 mg/kg GGF2	Vehicle only
	5 mg/kg GGF2	5 mg/kg GGF2	Vehicle only
	Vehicle only	Vehicle only	15 mg/kg GGF2

*—bilateral CN crush injury; **—glial growth factor 2.

## Data Availability

Data available on request.
